# HIV Partner Service Delivery Among Transgender Women — United States, 2013–2017

**DOI:** 10.15585/mmwr.mm6902a3

**Published:** 2020-01-17

**Authors:** Wei Song, Mesfin S. Mulatu, Shubha Rao, Guoshen Wang, Hui Zhang Kudon, Kevin O’Connor

**Affiliations:** ^1^Division of HIV/AIDS Prevention, National Center for HIV/AIDS, Viral Hepatitis, STD, and TB Prevention, CDC; ^2^Division of STD Prevention, National Center for HIV/AIDS, Viral Hepatitis, STD, and TB Prevention, CDC.

Transgender women* in the United States are disproportionately affected by human immunodeficiency virus (HIV) infection because of multiple factors, including stigma related to gender identity, unstable housing, limited employment options, and high-risk behaviors, such as sex work, unprotected receptive anal intercourse, and injection drug use, that tend to increase their vulnerability to becoming infected with HIV (*1*,*2*). In a recent meta-analysis of 88 U.S. studies conducted during 2006–2017, the mean estimated laboratory-confirmed prevalence of HIV infection among transgender women was 14.2%, and the mean self-reported prevalence estimate was 21.0% (*3*). The Ending the HIV Epidemic initiative calls for accelerating the implementation of evidence-based strategies in the right geographic areas targeted to the right persons to end the HIV epidemic in the United States (*4*). HIV partner services are effective strategies offered by public health workers to persons with a diagnosis of HIV infection (index persons) and their sex or needle-sharing partners (partners), who are notified of potential HIV exposure and offered HIV testing and related services. CDC analyzed HIV partner services data submitted by 61 health departments^†^ during 2013–2017. Among 208,304 index persons, 1,727 (0.8%) were transgender women. Overall, 71.5% of index transgender women were interviewed for partner services, which was lower than that for all index persons combined (81.1%). Among 1,089 transgender women named as partners by index persons, 71.2% were notified of potential HIV exposure, which was lower than that for all partners combined (77.1%). Fewer than half (46.5%) of notified transgender women partners were tested for HIV, and approximately one in five (18.6%) of those who were tested received a new diagnosis of HIV infection, slightly higher than for all partners combined (17.6%). Additional efforts are needed to effectively implement partner services among transgender women and identify those whose infection with HIV is undiagnosed, provide timely prevention and care services, reduce HIV transmission, and contribute to ending the HIV epidemic.

During 2013–2017, CDC funded 61 state and local health departments to implement comprehensive HIV prevention programs, including partner services. CDC analyzed HIV partner services person-level data for transgender women, identified using self-reported sex at birth and current gender identity. Data were stratified by age group, race/ethnicity, and U.S. Census region.^§^ Index persons are eligible for partner services if they live within the jurisdiction at the time of report. During partner services interviews, index persons can provide information about their sex or needle-sharing partners. Named partners are eligible for partner services if there is sufficient information to locate and notify them of their potential HIV exposure. Partners with newly diagnosed HIV infection are defined as those who test positive for HIV through partner services–initiated HIV testing and have no evidence of a previous diagnosis of HIV infection. Partners with previously diagnosed HIV infection should have evidence of an HIV diagnosis from cross-check with the health department surveillance system, review of laboratory reports, medical records, other available data sources (e.g., partner services database), or patient self-report. Data on index persons and partners were extracted from index person and partner information–specific databases; index persons could not be directly linked with their named partners. The outcomes for this analysis are the percentage of index transgender women interviewed for partner services and the percentage of transgender women partners notified and tested for HIV, and who newly or previously received a diagnosis of infection with HIV. Multivariate binomial regression was used to assess the association between index person or partner characteristics and partner services outcomes. However, because of the small number of partners with newly or previously diagnosed HIV infection, associations between partner characteristics and diagnosis of infection with HIV were not analyzed. SAS (version 9.4; SAS Institute) was used to conduct all analyses.

Among the 208,304 index persons reported to CDC during 2013–2017, 81.1% overall were interviewed for partner services (Table 1). Among all index persons, 1,727 (0.8%) were identified as transgender women, among whom 71.5% were interviewed for partner services. Compared with transgender women aged 13–24 years, those aged ≥35 years were less likely to be interviewed for partner services (adjusted prevalence ratio [aPR] for persons aged 35–44 years = 0.86; ≥45 years = 0.82). Compared with transgender women residing in the Northeast, those residing in the Midwest (aPR = 1.18) and in the South (aPR = 1.15) were more likely, and those residing in the West (aPR = 0.75) were less likely to be interviewed for partner services.

**TABLE 1:** Human immunodeficiency virus (HIV)-positive transgender women who were interviewed for partner services, by demographic characteristics — United States,* 2013–2017

Characteristic	All index persons			Index transgender women			
	Total, no.	Interviewed, no. (column %)	% Interviewed	Total, no. (%)	Interviewed, no. (column %)	% Interviewed	aPR (95% CI)
**Total**	**208,304**	**168,977 (100.0)**	**81.1**	**1,727 (100.0)**	**1,234 (100.0)**	**71.5**	**–**
**Age group (yrs)^†^**							
13–24	31,005	26,809 (15.9)	86.5	364 (21.1)	298 (24.2)	81.9	Reference
25–34	64,870	53,284 (31.5)	82.1	721 (41.8)	538 (43.6)	74.6	0.95 (0.89–1.01)
35–44	42,377	33,346 (19.7)	78.7	371 (21.5)	236 (19.1)	63.6	0.86 (0.78–0.94)**
≥45	62,029	48,827 (28.9)	78.7	269 (15.6)	162 (13.1)	60.2	0.82 (0.73–0.91)**
**Race/Ethnicity^§^**							
White, non-Hispanic	60,649	46,513 (27.5)	76.7	205 (11.9)	127 (10.3)	62.0	Reference
Black, non-Hispanic	88,878	76,198 (45.1)	85.7	890 (51.5)	694 (56.2)	78.0	1.09 (0.98–1.22)
Hispanic/Latino	42,460	34,417 (20.4)	81.1	453 (26.2)	301 (24.4)	66.4	1.09 (0.96–1.24)
Others, non-Hispanic	6,325	4,739 (2.8)	74.9	100 (5.8)	69 (5.6)	69.0	1.18 (1.00–1.14)
**U.S. Census region** ^¶^							
Northeast	26,658	23,442 (13.9)	87.9	410 (23.7)	301 (24.4)	73.4	Reference
Midwest	26,678	23,656 (14.0)	88.7	160 (9.3)	140 (11.4)	87.5	1.18 (1.08–1.28)**
South	110,039	95,961 (56.8)	87.2	596 (34.5)	497 (40.3)	83.4	1.15 (1.07–1.23)**
West	43,163	24,167 (14.3)	56.0	559 (32.4)	294 (23.8)	52.6	0.75 (0.67–0.83)**
U.S. dependent areas	1,766	1,751 (1.04)	99.2	2 (0.1)	2 (0.2)	100.0	–

**Abbreviations:** aPR = adjusted prevalence ratio for each binomial relationship controlling for other characteristics in the model; CI = confidence interval; MSA = metropolitan statistical area.

* Includes U.S. dependent areas of Puerto Rico and the U.S. Virgin Islands.

^†^ Because of missing/invalid data, records were excluded in the column “All index persons” for number of total (8,023; 3.9%) and number of interviewed (6,711; 4.0%) and in the column “Transgender women index persons” for number of total (2; 0.1%).

^§^ Because of missing/invalid data, records were excluded in the column “All index persons” for number of total (9,992; 4.8%) and number of interviewed (7,110; 4.2%) and in the column “Index transgender women” for number of total (79; 4.6%) and number of interviewed (43; 3.5%).

^¶^ U.S. Census regions (states and MSAs): *Northeast:* Connecticut, Maine, Massachusetts, New Hampshire, New Jersey, New York, New York City (New York), Pennsylvania, Philadelphia (Pennsylvania), Vermont, and Rhode Island. *Midwest*: Illinois, Chicago (Illinois), Indiana, Iowa, Kansas, Michigan, Minnesota, Missouri, Nebraska, North Dakota, Ohio, South Dakota, and Wisconsin; *South*: Alabama, Arkansas, Delaware, District of Columbia, Florida, Georgia, Atlanta (Georgia), Kentucky, Louisiana, Maryland, Baltimore (Maryland), Mississippi, North Carolina, Oklahoma, South Carolina, Tennessee, Virginia, Texas, and West Virginia. *West*: Alaska, Arizona, California, Los Angeles (California), San Francisco (California), Colorado, Hawaii, Idaho, Montana, Nevada, New Mexico, Oregon, Utah, Washington, and Wyoming; U.S. dependent areas: Puerto Rico and U.S. Virgin Islands.

** p<0.001.

Among partners identified in partner services interviews, 132,938 with sufficient information for follow-up were reported to CDC during 2013–2017 (Table 2), 102,500 (77.1%) of whom were notified. Transgender women partners with sufficient information for follow-up accounted for 1,089 (0.8%), among whom, 775 (71.2%) were notified of their potential HIV exposure. Compared with transgender women partners aged 13–24 years, those aged ≥25 years were less likely to be notified (aPR for 25–34 years = 0.88; 35–44 years = 0.79; ≥45 years = 0.77); compared with transgender women partners who were non-Hispanic white (white), those who were non-Hispanic black (black) were less likely to be notified (aPR = 0.89). Transgender women partners residing in the South and the West U.S. Census regions were more likely to be notified than those residing in the Northeast (aPR = 2.00 and aPR = 1.35, respectively).

**TABLE 2:** Partner notification services delivery among transgender women partners, by demographic characteristics — United States,* 2013–2017

Characteristic	All partners			Transgender women partners			
	Total, no.	Notified (column %)	% Notified	Total, no. (%)	Notified (column %)	% Notified	aPR (95% CI)
**Total**	**132,938**	**102,500 (100.0)**	**77.1**	**1,089 (100.0)**	**775 (100.0)**	**71.2**	–
**Age group (yrs)^†^**							
13–24	21,502	17,717 (17.3)	82.4	217 (19.9)	180 (23.2)	82.9	Reference
25–34	41,969	33,749 (32.9)	80.4	356 (32.7)	259 (33.4)	72.8	0.88 (0.81–0.95)^††^
35–44	22,936	17,957 (17.5)	78.3	195 (17.9)	123 (15.9)	63.1	0.79 (0.70–0.89)**
≥45	27,088	20,998 (20.5)	77.5	165 (15.2)	99 (12.8)	60.0	0.77 (0.68–0.88)**
**Race/Ethnicity^§^**							
White, non-Hispanic	38,622	29,528 (28.8)	76.5	245 (22.5)	193 (24.9)	78.8	Reference
Black, non-Hispanic	53,805	42,715 (41.7)	79.4	601 (55.2)	437 (56.4)	72.7	0.89 (0.81–0.97)^††^
Hispanic/Latino	24,593	19,615 (19.1)	79.8	172 (15.8)	102 (13.2)	59.3	0.93 (0.80–1.07)
Others, non-Hispanic	3,456	2,539 (2.5)	73.5	21(1.9)	10 (1.3)	47.6	0.90 (0.60–1.36)
**U.S. Census region** ^¶^							
Northeast	19,495	11,420 (11.1)	58.6	151 (13.9)	58 (7.5)	38.4	Reference
Midwest	14,291	8,185 (8.0)	57.3	26 (2.4)	14 (1.8)	53.8	1.43 (0.94–2.19)
South	71,459	65,640 (64.0)	91.9	766 (70.3)	629 (81.2)	82.1	2.00 (1.61–2.47)**
West	25,614	15,470 (15.1)	60.4	145 (13.3)	73 (9.4)	47.4	1.35 (1.03–1.76)^††^
U.S. dependent areas	2,079	1,785 (1.7)	85.9	1 (0.1)	1 (0.1)	100.0	–

**Abbreviations:** aPR = adjusted prevalence ratio for each binomial relationship controlling for other characteristics in the model; CI = confidence interval; MSA = metropolitan statistical area.

* Includes U.S. dependent areas of Puerto Rico and the U.S. Virgin Islands.

^†^ Because of missing/invalid data, records were excluded in the column “All partners” for number of total (19,443; 14.6%) and number of notified (12,079; 11.8%) and in the column “Transgender women partners” for number of total (156; 14.3%) and number of notified (114; 14.7%).

^§^ Because of missing/invalid data, records were excluded in the column “All partners” for number of total (12,462; 9.4%) and number of notified (8,103; 7.9%) and in the column “Transgender women partners” for number of total (50; 4.6%) and number of notified (33; 4.3%).

^¶^ U.S. Census regions (states and MSAs): *Northeast*: Connecticut, Maine, Massachusetts, New Hampshire, New Jersey, New York, New York City (New York), Pennsylvania, Philadelphia (Pennsylvania), Vermont, and Rhode Island. *Midwest*: Illinois, Chicago (Illinois), Indiana, Iowa, Kansas, Michigan, Minnesota, Missouri, Nebraska, North Dakota, Ohio, South Dakota, and Wisconsin; *South*: Alabama, Arkansas, Delaware, District of Columbia, Florida, Georgia, Atlanta (Georgia), Kentucky, Louisiana, Maryland, Baltimore (Maryland), Mississippi, North Carolina, Oklahoma, South Carolina, Tennessee, Virginia, Texas, and West Virginia. *West*: Alaska, Arizona, California, Los Angeles (California), San Francisco (California), Colorado, Hawaii, Idaho, Montana, Nevada, New Mexico, Oregon, Utah, Washington, and Wyoming; U.S. dependent areas: Puerto Rico and U.S. Virgin Islands.

** p<0.001.

^††^ p<0.05.

Among all 102,500 notified partners, 50.8% (52,071) were tested for HIV, among whom 9,146 (17.6%) received a new diagnosis of HIV infection (Table 3). Overall, 0.76% (775) of notified partners were transgender women, among whom 360 (46.5%) were tested for HIV; 67 (18.6%) of these women received a new diagnosis of HIV infection, and 18 (5.0%) had a previous diagnosis of infection with HIV. The highest testing percentages among transgender women partners were in those aged 25–34 years (52.5%), Hispanics/Latinos (51.0%), and residents of the Midwest (71.4%) Census regions (excluding U.S. dependent areas). Compared with transgender women partners who were white, those who were black were less likely to be tested for HIV (aPR = 0.83).

**TABLE 3:** Human immunodeficiency virus (HIV) testing and HIV positivity among transgender women partners, by demographic characteristics — United States,* 2013–2017

Characteristic	All notified partners				Notified transgender women partners					
	Notified, no.	Tested, no. (%)	Newly diagnosed HIV infection		Notified, no.	Tested, no. (%)	aPR (95% CI)	Newly diagnosed HIV infection		Previously diagnosed HIV infection
			No. (column %)	Row %				No. (column %)	Row %	No. (%)
**Total**	**102,500**	**52,071 (50.8)**	**9,146 (100.0)**	**17.6**	**775**	**360 (46.5)**	**—**	**67 (100.0)**	**18.6**	**18 (5.0)**
**Age group (yrs)^†^**										
13–24	17,717	10,580 (59.7)	1,769 (19.3)	16.7	180	90 (50.0)	Reference	20 (29.9)	22.2	6 (6.7)
25–34	33,749	18,094 (53.6)	3,075 (33.6)	17.0	259	136 (52.5)	1.03 (0.85–1.24)	23 (34.3)	16.9	9 (6.6)
35–44	17,957	9,690 (54.0)	1,725 (18.9)	17.8	123	56 (45.5)	0.90 (0.70–1.15)	10 (14.9)	17.9	2 (3.6)
≥45	20,998	10,838 (51.6)	2,258 (24.7)	20.8	99	49 (49.5)	0.95 (0.74–1.23)	9 (13.4)	18.4	1 (2.0)
**Race/Ethnicity^§^**										
White, non-Hispanic	29,528	15,607 (52.9)	2,520 (27.6)	16.1	193	95 (49.2)	Reference	14 (20.9)	14.7	2 (2.1)
Black, non-Hispanic	42,715	21,658 (50.7)	4,666 (51.0)	21.5	437	193 (44.2)	0.83 (0.69–0.99)**	38 (56.7)	19.7	13 (6.7)
Hispanic/Latino	19,615	10,240 (52.2)	1,374 (15.0)	13.4	102	52 (51.0)	0.97 (0.75–1.26)	12 (17.9)	23.1	3 (5.8)
Others, non-Hispanic	2,539	1,286 (50.6)	200 (2.2)	15.6	10	4 (40.0)	0.60 (0.25–1.46)	1 (1.5)	25.0	0 (0.0)
**U.S. Census region^¶^**										
Northeast	11,420	4,245 (37.2)	707 (7.7)	16.7	58	30 (51.7)	Reference	13 (19.4)	43.3	2 (6.7)
Midwest	8,185	4,342 (53.0)	884 (9.7)	20.4	14	10 (71.4)	1.31 (0.85–2.04)	6 (9.0)	60.0	0 (—)
South	65,640	34,122 (52.0)	6,465 (70.7)	18.9	629	286 (45.5)	0.91 (0.71–1.17)	38 (56.7)	13.3	14 (4.9)
West	15,470	8,294 (53.6)	978 (10.7)	11.8	73	33 (45.2)	0.78 (0.53–1.13)	9 (13.4)	27.3	2 (6.1)
U.S. dependent areas	1,785	1,068 (59.8)	112 (1.2)	10.5	1	1 (100.0)	—	1 (1.5)	100.0	0 (—)

**Abbreviations**: aPR = adjusted prevalence ratio for each binomial relationship controlling for other characteristics in the model; CI = confidence interval; MSA = metropolitan statistical area.

* Includes U.S. dependent areas of Puerto Rico and the U.S. Virgin Islands.

^†^ Because of missing/invalid data, records were excluded in the column “All notified partners” for number of notified (12,079; 11.8%), number of tested (2,869; 5.5%), number of newly diagnosed HIV (319; 3.5%) and in the column “Notified transgender women partners) for number of notified (114; 14.7%), number of tested (29; 8.1%), number of newly diagnosed HIV (5; 7.5%).

^§^ Because of missing/invalid data, records were excluded in the column “All notified partners” for number of notified (8,103; 7.9%), number of tested (3,280; 6.3%), number of newly diagnosed HIV (386; 4.2%) and in the column “Notified transgender women partners” for number of notified (33; 4.3%), number of tested (16; 4.4%), number of newly diagnosed HIV (2; 3.0%).

^¶^ U.S. Census regions (states and MSAs): *Northeast*: Connecticut, Maine, Massachusetts, New Hampshire, New Jersey, New York, New York City (New York), Pennsylvania, Philadelphia (Pennsylvania), Vermont, and Rhode Island. *Midwest*: Illinois, Chicago (Illinois), Indiana, Iowa, Kansas, Michigan, Minnesota, Missouri, Nebraska, North Dakota, Ohio, South Dakota, and Wisconsin; *South*: Alabama, Arkansas, Delaware, District of Columbia, Florida, Georgia, Atlanta (Georgia), Kentucky, Louisiana, Maryland, Baltimore (Maryland), Mississippi, North Carolina, Oklahoma, South Carolina, Tennessee, Virginia, Texas, and West Virginia. *West*: Alaska, Arizona, California, Los Angeles (California), San Francisco (California), Colorado, Hawaii, Idaho, Montana, Nevada, New Mexico, Oregon, Utah, Washington, and Wyoming; U.S. dependent areas: Puerto Rico and U.S. Virgin Islands.

** p<0.05.

## Discussion

This analysis found that the percentage of index transgender women interviewed by CDC-funded health departments was lower (71.5%) than that for all index persons combined (81.1%). There were also significant regional and age group differences among index transgender women interviewed. The percentage of transgender women partners notified of their potential HIV exposure (71.2%) was lower than that for all partners combined (77.1%), suggesting that there are missed opportunities to improve health of transgender women and to interrupt onward transmission of HIV.

Although 46.5% of transgender women partners were tested for HIV, this represented an improvement compared with the 35.6% ever testing and 10.0% past-year testing among transgender women found in an analysis of 2014–2015 Behavioral Risk Factor Surveillance data from 27 states and Guam (*5*) and was similar to the percentage of transgender women tested for HIV during the past 12 months (53.5%) through CDC-funded community-based organizations in three cities in 2008 (*6*). HIV testing is the gateway to other HIV-related services, and low rates of testing limit opportunities for timely linkage to care and prevention services (*7*).

Among transgender women partners who were tested, approximately one in five (18.6%) received a new diagnosis of HIV infection. This is consistent with an overall estimate of self-reported and laboratory-confirmed HIV prevalence of 18.8% among transgender women found in a meta-analysis of U.S. studies (*3*) and 19% pooled prevalence from 14 countries (*8*). Among transgender women partners with HIV-positive test results, 64.2% were aged ≤34 years, 56.7% were black, and 56.7% resided in the South. These findings are similar to those from the National HIV Surveillance System during 2009–2014, in which 72.6% of transgender women with HIV-positive test results were aged ≤34 years, 50.8% were black, and 42.8% resided in the South at the time of their diagnosis (*9*). Previous studies have attributed the higher levels of diagnosis of infection with HIV among transgender women, compared with those among other genders, to individual, social, and structural factors, including higher levels of sexual and drug use risk behaviors, gender and HIV-related stigma, homelessness, and mental health and substance use disorders (*1*,*2*).

The findings in this report are subject to at least four limitations. First, these analyses are based on HIV partner services program data reported from CDC-funded health departments and might not be generalizable to HIV partner services among all transgender women nationally. Second, the partners in the current analysis are those for whom sufficient information to be contacted by partner services programs was available and not all partners named by index persons. Third, the percentage of persons with newly diagnosed infection with HIV might be overestimated in jurisdictions that do not routinely check surveillance records to identify persons with previous diagnoses. Finally, health departments differ in implementation of partner services, which can contribute to varying data completeness and comparability.

Full and effective implementation of partner services programs is important to identify persons who are unaware of their HIV status. Partner services is a successful strategy for identifying persons with undiagnosed infection with HIV. However, the percentage of index person interview or partner notification for transgender women are lower than the national average for all genders combined. Approximately half of notified transgender women partners were tested for HIV. Efforts to address social and structural barriers to effective implementation of partner services among transgender women, including client concerns about compromised confidentiality and fear of negative impacts (e.g., abuse, stigmatization, medical mistrust, and abandonment), would improve partner services delivery in this disproportionately affected population (*2*,*10*). To that end, CDC has been supporting a variety of strategies, including conducting prevention research to identify evidence-based interventions that focus on transgender women, funding HIV prevention projects that prioritize transgender persons, and developing social media and marketing campaigns that promote HIV testing, prevention, and treatment among transgender persons (*1*). HIV prevention programs tailored to the needs of transgender women, particularly transgender women who are black, aged ≤35 years, and residing in the South, could help to reduce onward HIV transmission, increase linkage to HIV medical care and prevention, reduce HIV-related health disparities, and contribute to ending the HIV epidemic in the United States.

**Summary**

**What is already known about this topic?**

An overall estimate of prevalence of infection with human immunodeficiency virus (HIV) was 18.8% among transgender women based on a meta-analysis of studies in the United States conducted during 2006–2017.

**What is added by this report?**

During 2013–2017, 71.5% of index transgender women were interviewed for partner services, 71.2% of transgender women partners were notified of their potential HIV exposure, 46.5% were tested for HIV, and 18.6% received a new diagnosis of HIV-positivity.

**What are the implications for public health practice?**

Providing partner services to index transgender women and transgender women partners requires additional efforts to address the social and structural barriers unique to this population, provide timely prevention services, help reduce HIV transmission, and end the HIV epidemic in the United States.

## Footnotes

* Transgender persons are those whose current gender identity differs from their sex assigned at birth. In this analysis, transgender women included those who identified themselves as “male-to-female transgender” or those who identified “male” as their sex assigned at birth and “female” as their current gender.

† Fifty states, the District of Columbia, Puerto Rico, the U.S. Virgin Islands, and eight directly funded metropolitan statistical areas (MSAs) or specified metropolitan divisions: Baltimore, Chicago, Fulton County (Atlanta), Houston, Los Angeles County, New York City, Philadelphia, San Francisco.

§ U.S. Census regions (states and MSAs): *Northeast*: Connecticut, Maine, Massachusetts, New Hampshire, New Jersey, New York, New York City (New York), Pennsylvania, Philadelphia (Pennsylvania), Vermont, and Rhode Island. *Midwes*t: Illinois, Chicago (Illinois), Indiana, Iowa, Kansas, Michigan, Minnesota, Missouri, Nebraska, North Dakota, Ohio, South Dakota, and Wisconsin; *South*: Alabama, Arkansas, Delaware, District of Columbia, Florida, Georgia, Atlanta (Georgia), Kentucky, Louisiana, Maryland, Baltimore (Maryland), Mississippi, North Carolina, Oklahoma, South Carolina, Tennessee, Virginia, Texas, and West Virginia. *West*: Alaska, Arizona, California, Los Angeles (California), San Francisco (California), Colorado, Hawaii, Idaho, Montana, Nevada, New Mexico, Oregon, Utah, Washington, and Wyoming; U.S. dependent areas: Puerto Rico and U.S. Virgin Islands.
